# Impact of Exposure Uncertainty on the Association between Perfluorooctanoate and Preeclampsia in the C8 Health Project Population

**DOI:** 10.1289/ehp.1409044

**Published:** 2015-06-19

**Authors:** Raghavendhran Avanasi, Hyeong-Moo Shin, Verónica M. Vieira, David A. Savitz, Scott M. Bartell

**Affiliations:** 1 Environmental Health Sciences Graduate Program, University of California, Irvine, Irvine, California, USA; 2 Department of Public Health Sciences, University of California, Davis, Davis, California, USA; 3 Program in Public Health, University of California, Irvine, Irvine, California, USA; 4 Department of Epidemiology, and; 5 Department of Obstetrics and Gynecology, Brown University, Providence, Rhode Island, USA; 6 Department of Statistics, and; 7 Department of Epidemiology, University of California, Irvine, Irvine, California, USA

## Abstract

**Background:**

Uncertainty in exposure estimates from models can result in exposure measurement error and can potentially affect the validity of epidemiological studies. We recently used a suite of environmental models and an integrated exposure and pharmacokinetic model to estimate individual perfluorooctanoate (PFOA) serum concentrations and assess the association with preeclampsia from 1990 through 2006 for the C8 Health Project participants.

**Objectives:**

The aims of the current study are to evaluate impact of uncertainty in estimated PFOA drinking-water concentrations on estimated serum concentrations and their reported epidemiological association with preeclampsia.

**Methods:**

For each individual public water district, we used Monte Carlo simulations to vary the year-by-year PFOA drinking-water concentration by randomly sampling from lognormal distributions for random error in the yearly public water district PFOA concentrations, systematic error specific to each water district, and global systematic error in the release assessment (using the estimated concentrations from the original fate and transport model as medians and a range of 2-, 5-, and 10-fold uncertainty).

**Results:**

Uncertainty in PFOA water concentrations could cause major changes in estimated serum PFOA concentrations among participants. However, there is relatively little impact on the resulting epidemiological association in our simulations. The contribution of exposure uncertainty to the total uncertainty (including regression parameter variance) ranged from 5% to 31%, and bias was negligible.

**Conclusions:**

We found that correlated exposure uncertainty can substantially change estimated PFOA serum concentrations, but results in only minor impacts on the epidemiological association between PFOA and preeclampsia.

**Citation:**

Avanasi R, Shin HM, Vieira VM, Savitz DA, Bartell SM. 2016. Impact of exposure uncertainty on the association between perfluorooctanoate and preeclampsia in the C8 Health Project population. Environ Health Perspect 124:126–132; http://dx.doi.org/10.1289/ehp.1409044

## Introduction

Input parameter uncertainty in exposure estimates contributes to exposure measurement error, which can be described as the difference between an individual’s true exposure and the assigned exposure estimate (Armstrong 1998). The difference between true and assigned exposure can result from inaccuracies in measurement or model-based estimation of environmental chemical concentrations, biomarkers, time–activity patterns, and/or pharmacokinetics. Retrospective fate and transport model estimates may be particularly prone to inaccuracies, and integrating multiple models in the process of an exposure assessment can result in structural uncertainty, whereby uncertainty in one model gets propagated through the following models and can contribute more to the overall uncertainty than all of the individual uncertainties combined (Özkaynak et al. 2008). The use of surrogates for pollutant-level and participant-level spatiotemporal input data, such as modeled pollutant concentrations, self-reported activity patterns, or only one exposure biomarker per participant in certain situations, can be viewed as a type of exposure measurement error in the assessment (Bartell et al. 2004; Sarnat et al. 2010; Shin et al. 2014; Tsuchiya et al. 2012; Wu et al. 2013).

Exposure measurement error has been shown to introduce bias and random error in environmental epidemiological studies (Thomas et al. 1993), and the quality of exposure data has been identified as a major determinant of the validity of environmental epidemiology studies (Baker and Niewenhuijsen 2008; Rothman et al. 2008). Random exposure measurement error can bias the odds ratio and other epidemiological effect estimates, and also diminish the precision and power of the epidemiologic studies. As a result, it typically hampers the ability to detect an association between the exposure and adverse health effects (Armstrong 1998). Although there is a substantial literature on the potential impacts of exposure measurement error on epidemiologic studies, much of the literature relies on theoretical examples and/or simplified assumptions such as statistically independent measurement errors across participants (Carroll et al. 2006; Gustafson 2003; Zeger et al. 2000). Therefore, there is a need to characterize uncertainty in exposure estimates and in turn, to evaluate its potential impacts on reported epidemiological associations.

Residents of the Mid-Ohio Valley have been exposed to perfluorooctanoate (PFOA) since the 1950s, when large amounts (in the form of ammonium perfluorooctanoate—APFO) were released into the atmosphere and discharged into the Ohio River from a DuPont chemical facility, contaminating surrounding air, soil, surface water, and groundwater with PFOA. The primary exposure pathway for nearby residents was consumption of water contaminated by long-term air emissions, deposition on surface soil, and transport through the vadose and saturated zones to public and private wells; an additional water consumption pathway occurred via direct emissions into the Ohio River, contaminating downstream water supplies (Paustenbach et al. 2007). A series of epidemiologic studies have been conducted on PFOA and adverse health outcomes for participants in the C8 Health Project, a cross-sectional study that collected residential, occupational, and medical histories and serum samples in the contaminated region from 2005 through 2006 (Frisbee et al. 2009). We previously conducted retrospective PFOA exposure assessment for participants in the C8 Health Project, integrating several environmental fate and transport models, an exposure model, and a pharmacokinetic model to estimate air and water concentrations, personal exposures, and serum concentrations from 1951 through 2008 using individual residential histories, drinking-water sources, and tap-water consumption rates (Shin et al. 2011a, 2011b). These serum concentration estimates have been used subsequently in various epidemiological studies led by C8 Science Panel members to evaluate associations between PFOA exposure and various adverse health effects (C8 Science Panel 2013).

This retrospective exposure assessment included uncertainty in input parameters used in our PFOA fate and transport models. Out of many input parameters, the soil adsorption coefficient (K_d_) of PFOA, annual emission rates from the production facility, fraction of organic carbon (f_oc_) in the surface soil and unsaturated soil zones, and historical pumping rates of public water wells were previously identified as being influential and uncertain due to incomplete data (Shin et al. 2011a, 2011b). Uncertainty in these and other parameters can affect the accuracy of exposure estimates and, subsequently, the validity of epidemiological study results. However, it is unclear to what extent uncertainties in the exposure estimates threaten the validity of those study results and other epidemiological findings in this study population.

Critical features of our exposure model include a common exposure pathway for people using the same public water source, and linkage of personal residential histories with specific public water sources over time. These model features are important not only as drivers of PFOA exposure, because contaminated drinking water is thought to be the predominant exposure route for most participants (Shin et al. 2011b), but also as indications that exposure uncertainty is unlikely to be statistically independent across participants with the same water source, or across years for the same participant.

Savitz et al. (2012) reported a modest association between estimated serum PFOA concentrations in the year of pregnancy and preeclampsia; restriction to participants with highest quality residential history data strengthened the correlation between the observed and the estimated serum concentrations (Shin et al. 2011b), and also the observed epidemiological association (Savitz et al. 2012). Heavily influenced by the study by Savitz et al. (2012), a recent review of existing literature concluded that there is a probable link between exposure to PFOA and pregnancy-induced hypertension/preeclampsia (C8 Science Panel 2011). Given the borderline statistical significance and the fact that the association strengthens with more accurate exposure assignments, it is important to study the potential impact of inaccurate exposure assignments on that epidemiological association.

The objective of the present study is to evaluate the potential impact of systematic and random uncertainty in the estimated PFOA drinking-water and serum concentrations on the epidemiological association between PFOA exposure and preeclampsia. For each of the six public water districts (PWD) in the C8 Health Project, we generated multiple plausible year-by-year PFOA drinking-water concentrations via Monte Carlo simulation (for a range of 2-, 5-, and 10-fold uncertainty) and used these new water concentrations to estimate serum PFOA concentrations using the integrated exposure and pharmacokinetic model. In this analysis we evaluated the impact of uncertainty in the fate and transport models by specifying probability distributions directly for PFOA drinking-water concentrations instead of specifying distributions for each of the many input parameters in the models; hence, it can be considered a screening-level uncertainty analysis. This analysis focuses solely on uncertainty in PFOA drinking-water concentrations and does not consider uncertainty in individual-level parameters (drinking-water intake and pharmacokinetics).

## Methods

*Retrospective exposure assessment.* To estimate historical PFOA serum concentrations for participants in the C8 Health Project, we previously conducted a retrospective exposure assessment (Shin et al. 2011a, 2011b) which includes PFOA release assessment, integrated fate and transport modeling, dose reconstruction, and estimation of historical serum PFOA concentrations for each participant. The major steps in that exposure assessment are summarized in the following paragraph.

**Figure 1:**
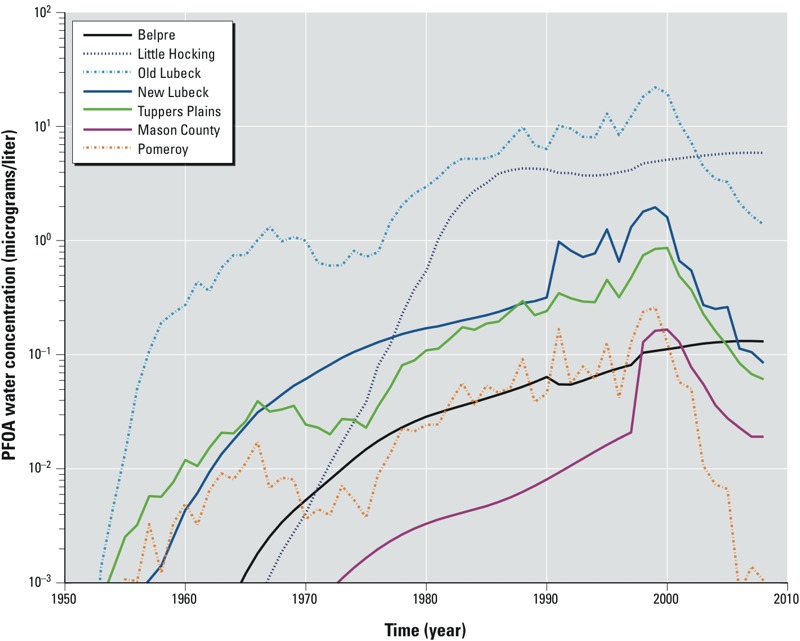
Estimated annual average PFOA water concentrations in the six public water districts (adapted from Shin et al. 2011a). Concentrations are shown in log (base 10) micrograms/liter.

First, historical PFOA emission rate estimates from the DuPont facility were obtained from a previous study conducted by Paustenbach et al. (2007). Second, we applied a suite of established environmental fate and transport models to estimate the concentrations of PFOA in the air, groundwater, and six municipal water supply wells around the facility for the years of 1951–2008. Input parameters of these environmental models include historical emission rate estimates, physicochemical properties of PFOA, and local meteorological and hydrologic data. The six PWDs that are involved in the C8 Health Project included the City of Belpre, Little Hocking Water Association, Tuppers Plains Chester Water District, the Village of Pomeroy Water District, Lubeck Public Services District, and Mason County Public Service District. Figure 1 shows the model-estimated PFOA water concentrations in the six PWDs over time from 1951 through 2008. Third, the estimated yearly air and water concentrations from environmental modeling were used to estimate historical PFOA exposures along with individual residential/work histories, demographic information (age, sex, body weight), standard exposure factors (air inhalation rate, drinking water ingestion rate), and historical pipe installation information of public water supply. Last, a single-compartment pharmacokinetic model was used to estimate year-by-year serum PFOA concentrations for each individual. Among all participants (*n* = 43,449), the Spearman’s rank correlation coefficient between the estimated and the 2005–2006 observed serum PFOA concentration (measured as a part of the C8 Health Project) was 0.67 (Shin et al. 2011b). Median estimated and observed serum concentrations in 2005–2006 were 13.7 and 23.5 ng/mL, respectively.

In the present analysis we did not change the first or second steps of the exposure assessment, but repeated the third and last steps many times using alternative water concentration estimates to gauge the potential impacts of uncertainties in PFOA drinking-water concentrations (uncertainties in the exposure assessment/pharmacokinetic models were not considered) on estimated serum concentrations and epidemiological results.

*Previous epidemiological analysis.* Using the estimated historical PFOA serum concentrations, Savitz et al. (2012) evaluated the associations between estimated PFOA serum concentrations at pregnancy and self-reported pregnancy related health outcomes, including preeclampsia among the C8 Health Project participants from 1990 through 2006 using generalized estimating equation regression models. There are 730 self-reported preeclampsia outcomes among the total 10,189 pregnancies. To address potential confounding, they adjusted for maternal age, parity, education, and maternal smoking status. The adjusted odds ratio (AOR) for the continuous exposure variable was 1.13 [95% confidence interval (CI) = 1.00, 1.28] for an interquartile range (IQR, 25th–75th percentile) of natural log–transformed (ln) PFOA serum concentration [IQR (lnPFOA) = 2.19 ln ng/mL].

We obtained approval (HS#2013-9421) from the Institutional Review Board at the University of California, Irvine, to work with the human subject data in this current study. We modified the original analysis by excluding 40 pregnancies of 25 mothers who had work histories in the DuPont PFOA production facilities. These participants might have had additional occupational exposure to PFOA before and during pregnancy, which sometimes exceeds the contribution from residential drinking-water ingestion. Excluding these pregnancies changes the AOR (95% CI) to 1.11 (0.99, 1.24) per IQR, with 725 preeclampsia outcomes among 10,149 pregnancies [IQR (lnPFOA) = 1.85 ln ng/mL].

*Monte Carlo simulation.* In the Monte Carlo uncertainty analysis, because public well water was a primary exposure route for our study population (Shin et al. 2011b), we selected year-by-year PFOA drinking-water concentrations for each of the six PWDs (output from the retrospective fate and transport model) as primary uncertain input parameters, and AOR for preeclampsia (output from the epidemiological model) as the output of Monte Carlo simulations. We assumed that PFOA drinking-water concentrations are lognormally distributed, because contaminant concentrations are non-negative (Limpert et al. 2001; Morgan et al. 1990).

For each of the six PWDs, we used the following equation to generate multiple simulated drinking water concentrations (*n* = 500, using Monte Carlo simulation) by multiplying the originally estimated average PFOA drinking-water concentrations (Shin et al. 2011a) by three multiplicative uncertainty factors, U1, U2, and U3:

C_i,j,k_ = C_0,i,j_ × U1_i,j,k_ × U2_i,k_ × U3_k_, [1]

where C_i,j,k_ is the simulated PFOA drinking-water concentration for a PWD *i* for a year *j* for the *k*th iteration. C_0,i,j_ is the previously estimated average PFOA drinking-water concentration for a PWD *i* for a year *j*. U1_i,j,k_ is the random uncertainty factor for a PWD *i* for a year *j* for the *k*th iteration not specific to any source and it varies the PFOA concentration by PWD by year by iteration. Ln U1_i,j,k_ follows a multivariate normal (MVN) distribution (corresponding to each year of exposure) with a mean of 0 for every year (represented as **0**, referring to a vector of zeroes of length 58), a correlation matrix of Σ, and a constant variance across years, σ^2^, that is, ln U1_i,j,k_ ~ MVN (**0**, Σ σ^2^). We chose off-diagonals of the correlation matrix to stipulate first-order autocorrelation of uncertainties across years, with an autocorrelation factor ϕ. Thus, sampled uncertainty factors for closer years are similar compared with those that are far apart. For example, the sampled PFOA concentrations that are 3 years apart will be correlated by a factor of ϕ^3^.

U2_i,k_ is the systematic uncertainty factor for a PWD *i* for the *k*th iteration due to mischaracterized PFOA transport in the unsaturated soil zone and groundwater aquifers within the groundwater catchment area of each PWD, so the PWD-specific uncertainty factor is applied during the time period when public water was a primary drinking-water source. Ln U2_i,k_ follows a normal distribution with a mean of 0 and variance of σ^2^, that is, ln U2_i,k_ ~ N (0, σ^2^). An example to describe U2 is the role of a parameter like the wind direction/speed. Any uncertainty in the wind direction/speed will affect the atmospheric transport and the deposition location of PFOA, systematically influencing each estimated PWD PFOA concentration for all years, but with a different magnitude and/or direction for each PWD. For example, mischaracterization of the wind speed and direction due to reliance on off-site meteorological data might be expected to systematically increase the PFOA deposition in some water districts for all years, and to decrease the PFOA deposition in other water districts for all years because a different prevailing wind direction would increase PFOA deposition rates for downwind water catchment basins but decrease deposition rates for other catchment basins.

U3_k_ is the global uncertainty factor for the *k*th iteration and includes systematic error that affects all PWDs and all years in the same way, such as systematic under- or overestimation of the PFOA emission rates. Ln U3_k_ follows a normal distribution with a mean of 0 and variance of σ^2^, that is, ln U3_k_ ~ N (0, σ^2^).

Because U1, U2, and U3 are generated independently of the original water concentration assignments C_0,i,j_, this model simulates additional classical (as opposed to Berkson) measurement error in the drinking water concentrations.

We repeated the analysis for four different hypothetical values of ϕ (which applies only to U1): 0, 0.5, 0.9, and 0.95 (chosen to represent a range starting with no correlation between adjacent years to a high correlation between adjacent years). The medians of U1, U2, and U3 are each set to 1 (giving equal probability for any randomly selected value to be higher or lower than 1), which corresponds to a ln mean of μ = 0. A range of ln variances (σ^2^): 0.13, 0.67, and 1.38, which corresponds to 95% probability intervals (PIs) of 2-, 5-, and 10-fold uncertainties, respectively (chosen to represent low, medium, and high levels of uncertainty), are simulated with the same value of σ^2^ used to specify the distributions of U1, U2, and U3. Thus, a total of 12 different Monte Carlo simulations were conducted corresponding to the various combinations of the ln variance parameter σ^2^ (0.13, 0.67, and 1.38, each applied to U1, U2, and U3) and ϕ (0, 0.5, 0.9, and 0.95, applied to U1 only).

MATLAB (Mathworks Inc., Natick, MA) and R (R Core Team 2015) were used to run Monte Carlo analyses. For each of the 500 Monte Carlo iterations, we applied simulated drinking-water concentrations to our integrated exposure and pharmacokinetic model to estimate serum concentrations and reanalyzed the association between newly simulated PFOA serum concentrations and the odds of preeclampsia occurrence. The AOR was computed per IQR of serum PFOA concentrations using multiple logistic regression, with recalculation of the IQR and a new regression for each Monte Carlo iteration.

We characterized overall uncertainty in the epidemiological association using the law of total variance: var(b) = E[var(b|X)] + var[E(b|X)], where b is the log odds parameter estimate and X is the collection of personal exposure estimates. The first term in the summation is the contribution of participant sampling uncertainty, and is estimated by the mean value of the log odds parameter variance across 500 iterations of the logistic regression. The second term in the summation is the contribution of exposure uncertainty, and is estimated by the variance of the log odds point estimate across 500 iterations. The standard error of the log odds is the square root of the total variance, and is used to produce 95% probability intervals summarizing the Monte Carlo simulation results. The percent contribution of exposure uncertainty to total uncertainty is given by var[E(b|X)]/var(b).

## Results

**Figure 2:**
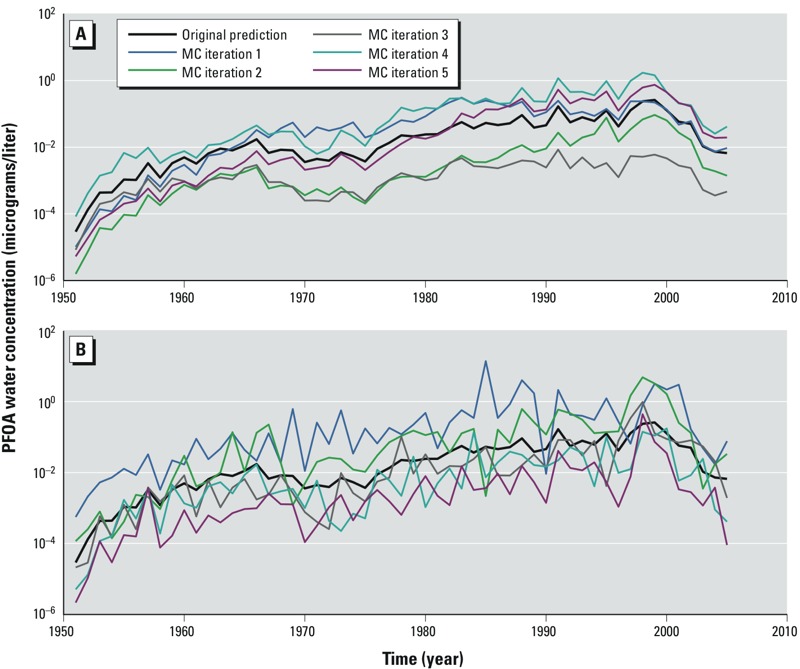
PFOA drinking water concentrations in Pomeroy PWD over time, comparing original estimates with Monte Carlo (MC) iterations using uncertainty factors with parameter values of σ^2^ = 0.13 and either (*A*) φ = 0.95 for high autocorrelation or (*B*) φ = 0 for no autocorrelation. Concentrations are shown in log (base 10) micrograms/liter

*Illustrative examples.* We begin by showing plots with results from individual iterations, using five Monte Carlo iterates as an illustrative example. Although five iterations are insufficient to generate a reliable sample for propagation of uncertainty, we find the plots helpful for visualizing the complex exposure patterns produced by our three-level uncertainty factors (U1, U2, and U3). To illustrate the combined effect of the three uncertainty factors, we randomly selected five sets of values (“iterations”) for U1, U2, and U3 from the appropriate probability distributions, and then computed PWD water concentrations for each iteration using Equation 1. Figure 2 shows PFOA concentrations in Pomeroy PWD (micrograms/liter) in log 10 scale over time for five iterations, with the upper panel representing the Monte Carlo simulation using uncertainty factors U1, U2, and U3, (ϕ = 0.95, σ^2^ = 0.13) and the lower panel representing the Monte Carlo simulation using uncertainty factors U1, U2, and U3, (ϕ = 0, σ^2^ = 0.13). The black line corresponds to the original estimated PFOA drinking-water concentrations, and the other five colored lines correspond to each of the Monte Carlo iterations obtained by multiplying the original PFOA concentration by the uncertainty factors. This example was chosen to visually show how the Monte Carlo simulation looks for the scenario when there is a low level of uncertainty in PWD concentration and high correlation versus no correlation between sampled uncertainty factors for adjacent years (U1). The Monte Carlo–simulated PWD PFOA concentration curves are smoother over time with ϕ = 0.95, as expected. ϕ = 0 corresponds to no correlation between the random values sampled (from the multivariate lognormal distribution U1) for adjacent years; for those simulations, the Monte Carlo simulation curves are more jagged. Adjacent-year PFOA drinking-water concentrations are expected to be correlated, and the PFOA concentration curves are smooth over time, because changes in PFOA flux to the surface soil will tend to be smoothed over time as PFOA travels through the subsurface into the groundwater table.

**Figure 3:**
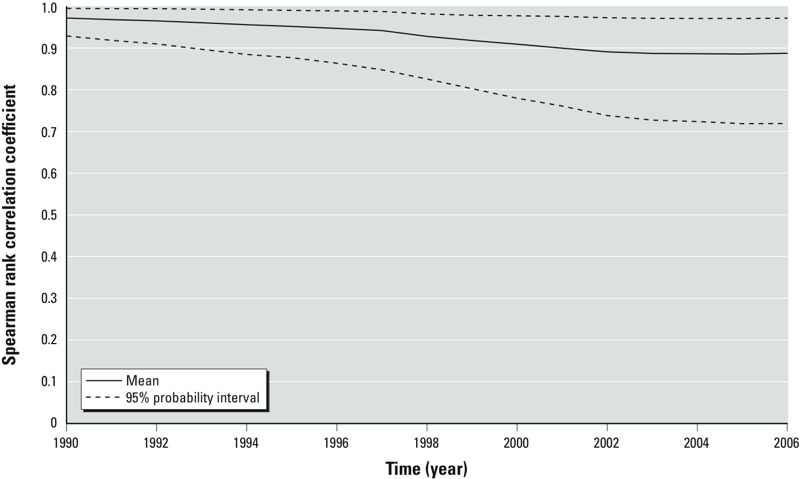
An example plot of the mean and the 95% probability interval of the correlation coefficient between the estimated serum concentrations for each Monte Carlo iterate and the original estimated serum concentrations, for all the participants, over time (U1, U2, and U3 with φ = 0.95, σ^2^ = 1.38).

*Full Monte Carlo simulation.* Next we present results for Monte Carlo simulation with 500 iterations for each of the 12 simulations (Table 1) using the full uncertainty model with U1, U2, and U3 (with σ^2^ = 0.13, 0.67, or 1.38, and ϕ = 0, 0.5, 0.9, or 0.95). Figure 3 is a plot of mean and 95% PI (the 2.5th and 97.5th percentiles) over 500 iterations of rank correlation between the simulated and original serum PFOA concentration estimates for all the Savitz et al. (2012) study participants from 1990 through 2006, for the Monte Carlo simulation using uncertainty factors U1, U2, and U3 (ϕ = 0.95, σ^2^ = 1.38). Although only one simulation is plotted here, it is the simulation with the highest impact of uncertainty on the serum prediction estimates (i.e., the other 11 simulations produce higher rank correlations).

**Table 1:** The mean and the 95% probability interval (PI) of the mean, median, and 25th and 75th percentile serum concentrations at birth (ng/mL), across 10,149 participants for each of the 12 Monte Carlo simulations (500 iterations per simulation).

Simulation	Mean (95% PI)	Median (95% PI)	25th percentile (95% PI)	75th percentile (95% PI)
Modified original	51.06	9.42	5.09	32.45
(σ^2^ = 0.13, φ = 0)	60.20 (27.07, 132.37)	9.73 (7.69, 13.15)	5.09 (4.94, 5.27)	36.35 (19.72, 71.53)
(σ^2^ = 0.13, φ = 0.50)	60.57 (25.80, 121.72)	9.73 (7.52, 12.56)	5.09 (4.91, 5.26)	36.74 (18.98, 64.36)
(σ^2^ = 0.13, φ = 0.90)	57.58 (27.27, 120.46)	9.56 (7.75, 12.17)	5.08 (4.95, 5.20)	34.67 (20.26, 62.98)
(σ^2^ = 0.13, φ = 0.95)	61.38 (26.65, 135.05)	9.66 (7.65, 12.67)	5.09 (4.91, 5.25)	36.11 (19.99, 68.27)
(σ^2^ = 0.67, φ = 0)	124.43 (17.73, 477.59)	11.27 (6.66, 23.65)	5.14 (4.88, 5.75)	55.16 (14.52, 181.99)
(σ^2^ = 0.67, φ = 0.50)	118.07 (15.19, 490.83)	10.89 (6.67, 26.49)	5.12 (4.81, 5.80)	54.57 (13.93, 209.57)
(σ^2^ = 0.67, φ = 0.90)	124.00 (16.82, 578.14)	10.61 (6.43, 21.34)	5.10 (4.78, 5.61)	53.73 (12.96, 218.77)
(σ^2^ = 0.67, φ = 0.95)	128.44 (12.85, 641.07)	10.77 (6.38, 26.59)	5.11 (4.80, 5.77)	55.79 (12.69, 222.65)
(σ^2^ = 1.38, φ = 0)	267.18 (14.73, 1595.08)	13.84 (6.14, 39.71)	5.19 (4.77, 5.92)	95.98 (11.42, 451.10)
(σ^2^ = 1.38, φ = 0.50)	455.98 (13.90, 2600.70)	14.27 (6.18, 57.83)	5.16 (4.78, 6.10)	102.68 (11.82, 620.82)
(σ^2^ = 1.38, φ = 0.90)	390.51 (11.50, 3075.62)	13.67 (5.86, 51.01)	5.14 (4.72, 6.11)	102.90 (11.19, 565.64)
(σ^2^ = 1.38, φ = 0.95)	396.66 (10.03, 2686.75)	12.47 (5.72, 35.71)	5.12 (4.69, 6.03)	83.63 (10.48, 433.71)
σ^2^ = Log variance of the uncertainty distributions U1, U2, and U3. φ = autocorrelation factor of uncertainty distribution U1.				

The mean, median, 25th–75th percentile serum concentrations at birth (nanograms per milliliter), across 10,149 participants were calculated, and their mean and 95% PI among 500 iterations are shown in the Table 1, along with those of the modified original analysis. The IQR of ln serum PFOA concentrations varied somewhat across the 500 iterations but generally remained the same order of magnitude as the original value of 1.85 ln ng/mL. For example, for σ^2^ = 0.13 and ϕ = 0, the 95% PI for the IQR was (1.38, 2.61) ln ng/mL, and for σ^2^ = 1.38 and ϕ = 0.95 the 95% PI for the IQR was (0.79, 4.33) ln ng/mL.

The mean and 95% PI for the AOR associating serum PFOA concentrations and preeclampsia for each simulation are shown in Table 2. The mean AOR in the 12 simulations ranged between 1.10 and 1.12. The percent contribution of exposure uncertainty to total uncertainty is tabulated in Table 3. Exposure uncertainty contributed anywhere between 5% and 31% to the total uncertainty in this analysis.

**Table 2:** The AOR (and 95% probability interval computed from the total standard error, which includes participant sampling variability and exposure uncertainty) when applying all uncertainty factors (U1, U2, and U3) simultaneously in Monte Carlo simulations.

φ	σ^2^		
	0.13	0.67	1.38
0	1.11 (0.99, 1.25)	1.11 (0.98, 1.26)	1.12 (0.97, 1.28)
0.5	1.11 (0.99, 1.25)	1.11 (0.98, 1.26)	1.11 (0.97, 1.27)
0.9	1.11 (0.99, 1.24)	1.11 (0.98, 1.25)	1.10 (0.96, 1.27)
0.95	1.11 (0.99, 1.25)	1.11 (0.97, 1.26)	1.10 (0.96, 1.26)
The AOR (and 95% confidence interval computed from participant sampling variability only) using the original exposure assignments is 1.11 (0.99, 1.24). σ^2^ = Log variance of the uncertainty distributions U1, U2, and U3. φ = autocorrelation factor of uncertainty distribution U1.			

**Table 3:** Percent contribution of participant exposure uncertainty to the total uncertainty for the combined effect of participant sampling variability and exposure uncertainty.

φ	σ^2^		
	0.13	0.67	1.38
0	5%	18%	29%
0.5	5%	19%	30%
0.9	5%	19%	31%
0.95	5%	21%	30%
σ^2^ = log variance of the uncertainty distributions U1, U2, and U3. φ = autocorrelation factor of uncertainty distribution U1; not applicable to uncertainty factors U2 or U3.			

## Discussion

Although incorporating autocorrelated and shared uncertainty in our water concentration estimates produced a highly variable set of plausible serum PFOA concentrations, it had less impact on the rank order of estimated serum PFOA concentrations during pregnancy. Moreover, these changes in estimated serum PFOA had a negligible impact on the mean AOR for preeclampsia and only modestly increased its total standard error, likely because the regression is more sensitive to the rank order of participant exposures than it is to absolute exposure assignments. The existing epidemiological literature suggests that adding independent, nondifferential classical exposure measurement error will tend to bias the effect estimate towards the null hypothesis (Armstrong 1998). However, we observed no substantial bias in our Monte Carlo simulations. This may be attributable to our focus on potential errors in characterizing PWD water concentrations, which are shared exposure sources, rather than simulating independent exposure measurement errors. As a brief test of that explanation, we ran two additional simulations without U1, U2, or U3, but now adding a new lognormal uncertainty factor for individual drinking-water ingestion rates, with 10-fold and 100-fold uncertainty (100 iterations each). Mean AORs in these simulations were 1.09 and 1.07, respectively, indicating greater sensitivity of the epidemiological results (the original AOR was 1.11) to independent exposure errors than to the shared exposure errors of the primary analysis shown in Table 2. The weak association between PFOA and preeclampsia may also make it appear less sensitive to both shared and independent exposure uncertainties (e.g., a change of the AOR from 1.11 to 1.07 appears small but actually constitutes a 35% decrease in the log odds parameter). PFOA water concentrations in the contaminated region differed by several orders of magnitude across PWDs and across years (Shin et al. 2011a), which may explain why perturbing the exposure estimates with as much as 10-fold uncertainty contributed only modestly to the total standard error and negligibly to bias. Indeed, using regression calibration (Rosner 2010) treating the Monte Carlo simulation as a simulated reproducibility study and assuming independent measurement errors across participants, we computed for the simulation with ϕ = 0.95 and σ^2^ = 1.38 an intraclass correlation coefficient of *r*_1_ = 0.25 and a corrected AOR of 1.72 (95% CI: 1.04, 2.87). The independence assumption is clearly unwarranted here, but this exercise illustrates that potential inaccuracies in our historical water concentration estimates may pose a far lesser threat to the validity of previously published epidemiological associations between PFOA and preeclampsia in the C8 Health Study than suggested by traditional models for exposure measurement error.

At the selected exposure uncertainty variances (σ^2^), varying the autocorrelation parameter (ϕ) had little impact on the output AOR distribution, only slightly increasing the total standard error. Although the direction of the effect is reasonable because a multi-year increase or multi-year decrease in water concentrations is more likely with higher autocorrelation and produces a larger change in serum concentration than a mix of yearly increases and decreases, we expected the total standard error to be more strongly affected by this parameter than it was. This is somewhat reassuring, considering that it is more difficult to interpret and choose a reasonable value of ϕ than σ^2^.

The contribution of correlated exposure uncertainty to the overall uncertainty in an epidemiological analysis of PFOA exposure and preeclampsia is estimated here. Traditional confidence intervals only account for participant sampling variance, not the effects of exposure uncertainty. In this specific PFOA exposure assessment–environmental epidemiology analysis, fate and transport model uncertainty seems to contribute only modestly to the overall uncertainty in the relationship between PFOA exposure and preeclampsia. Although these results cannot be generalized to other settings, the methods could be applied to other epidemiological analyses including studies of PFOA and other health effects in this population. This may be particularly important in weighing disparate findings from studies that use different methods of exposure assessment (e.g., fate and transport models, questionnaires, and/or biomarkers). Although meta-analysis provides a method for combining disparate study findings, it traditionally weighs studies only by their estimated parameter variances (i.e., sampling variability) and does not address the quality of exposure assessment or other study design characteristics.

Drinking-water ingestion is a major exposure route (vs. inhalation or dermal exposure) for our study population in all years, except for the participants who consumed water from Little Hocking before 1974 and those who consumed water from Belpre before 1990 (Shin et al. 2011b). Given this, and the fact that the epidemiological analysis included pregnancies occurring only between 1990 and 2006, we chose to model uncertainty only for the drinking-water concentrations in this analysis, not perturbing the original inhalation exposure estimates for each Monte Carlo iteration. Private well water has been used by participants in the study over their residential history in the area and can be a potential source of uncertain PFOA exposure to the participants. However, only 9.6% of the Savitz et al. (2012) study participants had at least one source of private water consumption between the years 1985 and 2006. Therefore, we did not consider the uncertainty in the private well PFOA concentrations in our analysis because we deemed it to be negligible compared with the PWD PFOA contribution to the total exposure. Another relatively minor source of PFOA exposure is the consumption of vegetables that were either grown locally or home grown; however, due to the sparseness of data specific to the individual participant vegetable consumption, the original model did not consider this route of exposure (Shin et al. 2011a). We also did not assess independent sources of error such as individual variations/uncertainty in ingestion rates and pharmacokinetics of PFOA. These uncertainties are likely to produce Berkson-like error structures in the individual exposure assignments (Berkson 1950; Heid et al. 2004), because group-level pharmacokinetic and water ingestion rates were assigned in the absence of individual-level data. Incorporation of these components into the uncertainty analysis would likely cause an increase in the apparent contribution of exposure uncertainty to uncertainty in the epidemiological findings.

Our uncertainty analysis explores the impact of changing the original PFOA exposure assignments by simulating additional measurement error, but it does not “correct” or “adjust” for errors in exposure assignment. Regression calibration (Rosner 2010) can be used to correct AORs to account for a simple exposure measurement error structure, but would have to be adapted for use with complex simulations such as our setting. Regression calibration includes three important assumptions that are not valid in our study: *a*) The measurement errors are normally distributed, *b*) the errors are statistically independent of the surrogate exposure and independent across individuals, and *c*) the other covariates in the regression model are measured without error. In our study, the measurement error components are lognormally distributed and strongly correlated among individuals with the same water source, and covariates such as smoking status were likely measured imperfectly due to the use of self-reports.

## Conclusions

The Monte Carlo uncertainty analysis described here can be considered a screening-level uncertainty analysis because we have characterized uncertainty in the environmental model estimated PWD PFOA concentrations as a surrogate for hundreds of parameters in the suite of fate and transport models used to estimate the PWD PFOA concentrations. Using separate U1, U2, and U3 uncertainty components allows for specification of correlations in exposure measurement errors across years and across individuals with shared exposure sources, in contrast to standard epidemiological models that assume independence of the measurement errors (Armstrong 1998; Rosner 2010). Due to the complexity of this particular suite of fate and transport models, which take days to weeks to run for a single set of input parameters, a parameter-based Monte Carlo uncertainty analysis would require a prohibitive amount of computer time. Our screening-level assessment suggests that correlated exposure measurement error may produce dramatic changes in PFOA serum estimates yet contribute only modestly to overall uncertainty regarding the epidemiological association between PFOA and preeclampsia. As a next step, exploring the impact of individual-level uncertainties in the exposure assessment and pharmacokinetic model will provide more insight regarding the effects of exposure uncertainty on this epidemiological association. Future epidemiological analyses might benefit from simulation studies or other techniques for evaluating the impacts of uncertainties in complex exposure models.
